# Coronary Plaque Characterization From Optical Coherence Tomography Imaging With a Two-Pathway Cascade Convolutional Neural Network Architecture

**DOI:** 10.3389/fcvm.2021.670502

**Published:** 2021-06-16

**Authors:** Yifan Yin, Chunliu He, Biao Xu, Zhiyong Li

**Affiliations:** ^1^School of Biological Science and Medical Engineering, Southeast University, Nanjing, China; ^2^Department of Cardiology, Nanjing Drum Tower Hospital, Nanjing, China; ^3^School of Mechanical, Medical, and Process Engineering, Queensland University of Technology, Brisbane, QLD, Australia

**Keywords:** optical coherence tomography, convolutional neural network, plaque characterization, cascaded structure, two-pathway architecture

## Abstract

**Background:** The morphological structure and tissue composition of a coronary atherosclerotic plaque determine its stability, which can be assessed by intravascular optical coherence tomography (OCT) imaging. However, plaque characterization relies on the interpretation of large datasets by well-trained observers. This study aims to develop a convolutional neural network (CNN) method to automatically extract tissue features from OCT images to characterize the main components of a coronary atherosclerotic plaque (fibrous, lipid, and calcification). The method is based on a novel CNN architecture called TwopathCNN, which is utilized in a cascaded structure. According to the evaluation, this proposed method is effective and robust in the characterization of coronary plaque composition from *in vivo* OCT imaging. On average, the method achieves 0.86 in F1-score and 0.88 in accuracy. The TwopathCNN architecture and cascaded structure show significant improvement in performance (*p* < 0.05). CNN with cascaded structure can greatly improve the performance of characterization compared to the conventional CNN methods and machine learning methods. This method has a higher efficiency, which may be proven to be a promising diagnostic tool in the detection of coronary plaques.

## Introduction

Cardiovascular disease remains to be the leading cause of morbidity and mortality globally. Acute events, such as heart attack and stroke, are usually triggered by the development of plaque rupture/erosion and subsequent thrombus formation. It has been shown that plaque components and morphology are the main factors in the determination of plaque stability (1–3). A coronary plaque normally consists of three different tissue types: fibrous, calcified, and lipid-rich, with mixed forms appearing in many cases. Plaques that have a large lipid core and a thin fibrous cap are more prone to rupture, whereas plaques containing calcification tend to be stable (4). Therefore, to prevent the acute cardiovascular events, it is crucial to develop strategies to characterize plaque morphology and components so that the vulnerable plaques can be identified at an early stage to reduce the risk of rupture. Based on the analysis of the reflected light, optical coherence tomography (OCT) has a high resolution. Its axial resolution can reach to 12–18 μm (5), which is a promising diagnostic technique in interventional cardiovascular imaging. High-resolution cross-sectional images of coronary artery can be seen *via* OCT, which allow us to observe the plaque components and morphology directly and distinguish different plaque types. The expert consensus document indicates that the fibrous tissue appears as uniform high-signal regions, the calcified tissue appears as uneven, low-signal regions with clear boundaries, whereas the lipid tissue is the low-signal region with blurred boundaries in OCT (6). In current clinical application, vascular lumen segmentation and characterization of plaque components are performed manually, which is laborious and time-consuming. Moreover, the accuracy is dependent on the experience of the observers. Therefore, developing a fast, accurate, and reliable method for an automatic characterization of atherosclerotic plaque composition is crucial, which can reduce the time and cost of analyzing data and avoid human interference errors, and thus can improve risk assessment of patients with coronary artery diseases.

Levitz et al. (7) published a study demonstrating that atherosclerotic plaque characterization by OCT could be done by measuring the back-scattering and attenuation coefficients (8), which enhanced the differentiations between the fibrous, calcified, and lipid tissues. The preliminary data indicated that differences in scattering properties may exist between the normal and the atherosclerotic plaques and that optical scattering properties provided by OCT can contribute to plaque characterization. After that, more optical parameters were introduced to characterize components of plaques in OCT studies (9–13). Although optical properties are good representations of various intracoronary tissues, detailed information regarding plaque morphology is required for better recognition of various plaque types. To utilize more texture features, machine learning methods have been applied to plaque characterization. Wang et al. (14) proposed a method for semiautomatic segmentation of calcified plaques with the morphology operation. Ughi et al. (15) combined the attenuation coefficients and Gray-Level Co-occurrence Matrix (GLCM) (16) to obtain texture features and utilized the random forest (17) model to classify the test set and compared the result with the manually segmented ground truth. The overall classification accuracy was 81.5% with a feasibility of 76.5%. The accuracy for each type was 89.5% for fibrotic tissue, 72.1% for calcium, and 79.5% for lipid-rich tissue. Athanasiou et al. (18) used K-means (19) to divide calcified tissue and applied GLCM and local binary pattern (20) to characterize texture features. Rico-Jimenez et al. (21) applied least square optimization strategy to estimate the depth of plaque. Our group (22) also presented the characterization of atherosclerotic plaque components based on the machine learning methods. Although these methods have shown a better performance compared to the methods based on attenuation coefficients, the features extracted by algorithm cannot completely describe all the global morphological information, thus leading to classification noise.

Convolutional neural networks (CNNs) (23) have shown remarkable successes in image processing tasks, such as image classification, facial identification (24), and object detection (25). In terms of OCT, Gessert et al. (26) developed a CNN model to detect images with calcified plaques. Kolluru et al. (27) implemented a CNN model consisting of two convolutional and max-pooling layers and applied a fully connected conditional random field (CRF) as a post-processing step to improve classification sensitivity. Yong et al. (28) proposed a linear-regression CNN model to segment the lumen in OCT images automatically. Lee et al. (29) implemented SegNet and Deeplab v3+ for plaque characterization in terms of pixel-wise and A-line-based classifications. Our group also presented calcium classification on the OCT pullback with 3D deep neural networks (30). Gharaibeh et al. (31) used deep learning and transfer learning methods to analyze the calcification components in OCT images. Li et al. (32) proposed a deep residual U-Net network for the segmentation of vulnerable plaque components in coronary OCT. Athanasiou et al. (33) proposed a patch-based CNN network to characterize plaque components. Previous studies on deep learning-based plaque characterization mainly fall into two categories: one is utilizing segmentation networks to learn and evaluate OCT images directly, such as FCN, SegNet, etc. However, this method requires a huge training dataset. The other way is using a patch-based CNN network to achieve the segmentation goal by classifying small patches, but some position and boundary information may be lost, and the evaluating efficiency is low because the networks need to characterize each pixel by classifying a patch. Most patch-based CNN models realize segmentation function by predicting the M × M patch centered on that pixel; the segmentation results are determined by the classification category of the M × M patch. Therefore, when evaluating the whole OCT image by normal patch-based CNN models, the experts need to make patches for all the pixels in the region of interest and classify them with CNN models. This means the CNN models have to classify thousands of patches when evaluating an OCT image.

Therefore, in this study, we designed a patch-based CNN model to analyze the OCT dataset of coronary arteries to characterize three plaque components automatically: fibrotic, calcified, and lipid-rich tissues. Three trained observers provided segmentations for each OCT image, and the selected regions were divided into patches with different sizes. All the patches were put into a CNN model to obtain an automated classifier of atherosclerotic plaques. For testing, the performance of the model was subsequently evaluated against the testing set manually segmented by a trained OCT observer, which includes 10 samples for three plaque components, respectively, 30 in total. To convince the reliability of testing results, these samples were selected from 10 different pullbacks and 3 different patients, which were not used in the training period. This is the first study that Two-Pathway Cascade CNN Architecture is applied into the field of coronary plaque characterization from OCT imaging. Compared with other patch-based CNN models, the method achieved a more accurate result than the methods published in the literature especially for the calcified plaques. The time complexity is also significantly reduced by using convolutional output layer, which makes the method more clinically realistic. Several novel strategies on CNN architectures and training process were utilized to avoid the weakness of patch-based CNN model, and demonstrated the effectiveness with statistical methods.

## Methods

### Data Collection

All the coronary OCT imaging data were collected from Nanjing Drum Tower Hospital. The whole imaging procedure was performed using the C7XR OCT imaging system (St. Jude Medical, Minneapolis, MN, USA) connected to a C7 Dragonfly intravascular imaging catheter (St. Jude Medical). During imaging, the catheter was inserted into the patient's coronary artery. By rotating the probe inside the catheter and pulling it backwards, the images inside the vessel were acquired. The light source emits an optical signal and the detector receives the interference signal produced by the signal light backscattered by the vessel and the reference light reflected by the reflector, then the system further processes the signal to obtain a scan line signal (A- line). The C7XR OCT has 504 scan A-lines per blood vessel section. In one scan, the C7XR OCT can obtain a 271-frame blood vessel image in polar representation with the resolution of 976 × 504 pixels in gray scale. For easier observation, all the images were transformed into Cartesian space with 1,024×1,024 pixels, and the B-scan is color-mapped to RGB using a colormap for visualization contrast.

**Figure 1:**
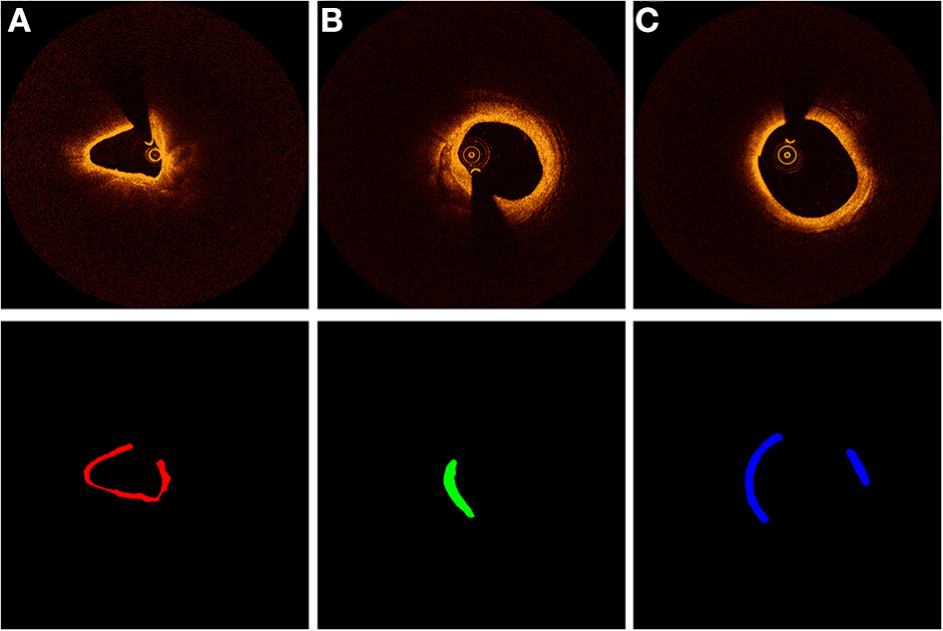
Characterization of coronary plaque components from OCT images. Different colors represent different plaque composition: **(A)** red: fibrous. **(B)** green: calcified, **(C)** blue: lipid.

For ground-truth annotation, three experienced observers firstly excluded images with stenting cases. To avoid plaques with similar patterns containing in the dataset, we extracted one frame in each adjacent three frames for dataset making. All the selected images were segmented manually. To ensure the reliability of dataset, all images were separated randomly to three observers for manual segmentation. After the segmentation, they exchanged the segmented OCT images and double-checked the results. When disagreement occurred, the final segmentation results were confirmed by an interventional cardiologist. Figure 1 shows the template of an example of the manually segmented plaque compositions.

In total, the dataset included 2,000 images from 31 patients, and 2,000 regions of interest were extracted from these images. Fibrous tissue and lipid tissue comprised 40% of the whole dataset, respectively, and calcified tissue occupied 20%. One sixth of the dataset were used to validate and the rest were used to train. The regions of interest in the training images (corresponding to color regions in the template) were sliced into the size of 65 × 65 × 3 and 33 × 33 × 3 pixels. Different patches (e.g., 17 × 17 × 3 or 83 × 83 × 3) were also tested, but 65 × 65 × 3 and 33 × 33 × 3 achieved the best performance. A smaller size cannot obtain location features in the training period while a larger size introduces more noise that may affect the training results. All the patches were labeled according to their corresponding color in the template. Here, we used patches with RGB channels for CNN training to guarantee that the images for prediction are in the same condition with those for manual analysis.

### Data Augmentation

From the number of patches belonging to each label, we discovered that patches belonging to calcified tissue were the least. Seriously, data imbalance can affect the training result of the CNN model. To deal with the problem of imbalanced data, the often-used approach is to modify loss function or use data augmentation. In our cases, modifying loss function did not perform efficiently because there was a considerable gap in the amount of data in each category. Thus, we used data augmentation to the calcified patches to enable the quantity of patches in each label to be strictly equal.

A certain number of calcified patches were randomly chosen for data augmentation. We applied flipping along the *x* and *y* directions on the chosen patches. In total, the final patches dataset consisted of 120,000 patches with equal quantity in each label. We split off an independent validation set of 20,000 patches to detect the learning condition of the CNN model during the training process and the others were considered as the training set. To further reduce the impact of imbalanced dataset, two-phased training strategy was utilized in the training process, which is introduced in section Segmentation Results.

### CNN Approach

Like most CNN-based segmentation models, we replaced the calculation of each pixel by predicting the M × M patch centered on that pixel. Thus, the input of CNN model was M × M patches with different types. By applying a series of convolutional layers, CNN had the ability to extract complex features. More detailed information of patches can be learned by treating the output feature maps of the previous convolutional layer as the input of the subsequent. To obtain non-linear features from the input, ReLU (34) was utilized as an activation function. A normalization process was also added after the activation function to bring mean value and variance close to zero and one, respectively. The normalization process can be summarized by the following formula:

$$x_{n}\;=\;\frac{x-\mu }{\sqrt{\sigma^{2}+t}}$$

Here, *x*_*n*_ and *x* represent the feature maps after and before the normalization process, respectively, and μ and σ^2^ are the mean and variance of the feature maps. *t* is a small constant to avoid division by zero.

Due to the lack of training examples in our study, the model was vulnerable to over-fitting. Accordingly, we used Dropout (35) regularization method to overcome it. When computing the hidden layer of CNN, dropout masks the input or hidden unit with a certain probability. In this case, the prediction of CNN did not only rely on a small part of weights in the entire network.

In conventional CNN models, for classification purposes, the output layer is typically fully connected, which is inefficient in segmentation. To perform a prediction of the segmentation labels, we replaced the fully connected layer by a convolutional output layer. The number of kernels in the layer equaled to the number of labels (four in our case, the background was also included). The output of kernels was considered as the final estimation of CNN model, which can be normalized by SoftMax function as follows:

$$Softmax(a)=\frac{exp(a)}{\sum_{i}exp(a_{i})}$$

Here, *a* is the output vector of convolutional output layer. Each element of *w* = SoftMax(*a*) is limited to the range of (0, 1) and the sum equals to 1. Thus, *w* is regarded as the probability distribution of all labels of the patches. The CNN approach performs the segmentation by assigning the label with largest probability to each pixel. By placing the convolutional output layer at the end, when doing segmentation tasks, the speed of testing was 40 times faster than the conventional CNN model. More details are shown in section Implementation Details.

### Two-Pathway CNN Architecture

To find the most appropriate CNN architecture for the segmentation task, we constructed a new CNN model instead of applying transfer learning because the state-of-the-art CNN architectures typically use large images as their training set (about 270 × 270 in most cases). Two-pathway CNN (TwoPathCNN) (36) was introduced to extract features. Different from the straight structure of conventional CNN networks, TwoPathCNN is made of two streams: a pathway with several convolutional layers in smaller receptive fields (local pathway) and the other with a convolutional layer in a larger receptive field (global pathway). After the convolution operation, a concatenation layer combined the feature maps from two streams together. In this architecture, the prediction of labels can be influenced by two aspects: the visual details of the region and the general features.

**Figure 2:**
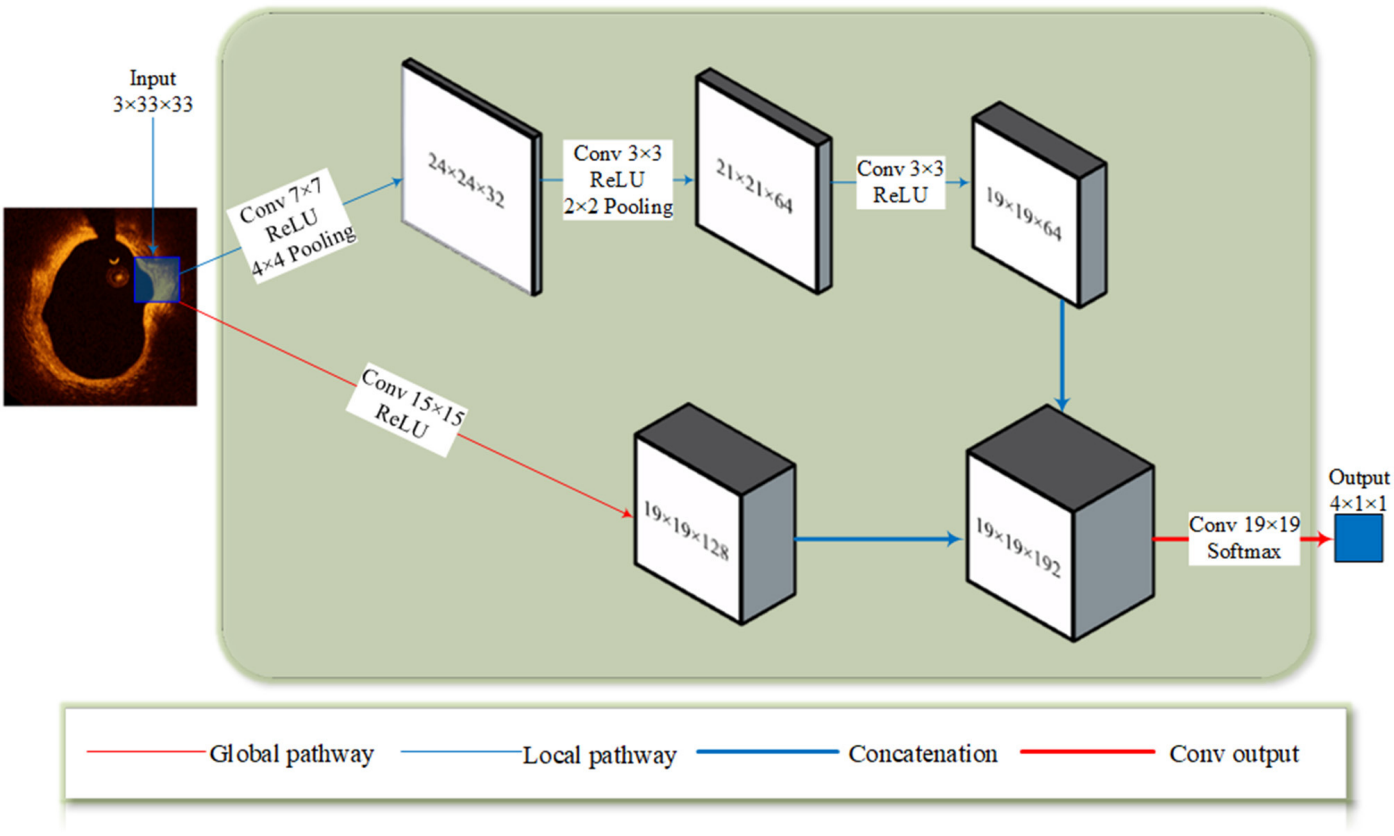
The TwoPathCNN architecture. The input patch goes through a global pathway (marked with a thin red line) and a local pathway (marked with a thin blue line) with convolution. The feature maps produced by two pathways are concatenated together (marked with a thick blue line) and it goes through a convolutional output layer (marked with a thick red line) to obtain the prediction.

The full architecture is illustrated in Figure 2. To satisfy the concatenation, the size of the top hidden layers of both pathways must be the same. The local pathway contains three convolutional layers with 7 × 7 and 3 × 3 kernels. The global pathway contains one convolutional with 15 × 15 kernels. In this network, more feature maps were extracted from the global pathway.

### Cascaded Structure

Single CNN structure predicts segmentation label according to each individual patch, lacking the information of position. In response to this shortcoming, the segmentation method in literatures often define a conditional random field (37, 38) to simulate the relationship between each label from a spatial perspective, and the final prediction is influenced by both the original prediction and segmentation labels in the vicinity.

**Figure 3:**
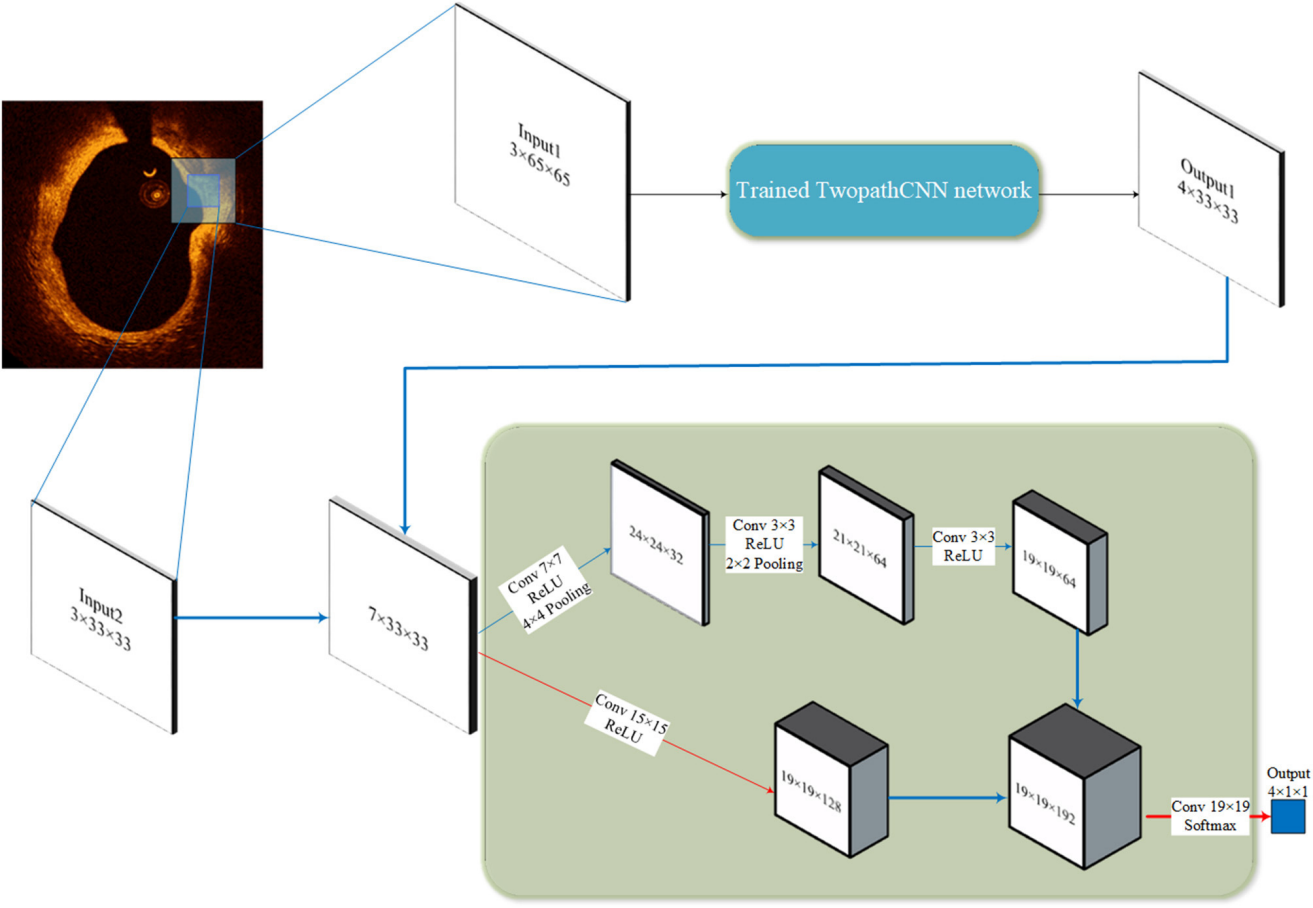
Cascaded structure. The 65 × 65 patches go through the trained TwopathCNN network (shown in Figure 2), and the results are concatenated with the 33 × 33 patches to build new training data.

In our study, the cascaded structure was applied to make a connection between CNN networks with different input sizes. As Figure 3 illustrated, the output of the first CNN is concatenated with the patches in a smaller size, the output and the patches both comprise the input to the second CNN. Thus, the input of the second CNN is the feature maps with seven channels. This kind of structure can overcome the ignorance of the relative position by regarding the probability distribution of labels of surrounding pixels as a prerequisite to the prediction. Compared to conditional random field, this method takes the advantage in the consuming of computational resource, which is important when dealing with large amounts of OCT data in clinical diagnosis.

### Lumen and Guidewire Line Segmentation

Due to the limitation of the tissue penetration of OCT (about 1–1.3 mm) (39), OCT may not be able to correctly display the region outside the region far away from the lumen border; therefore, the region of interest (vascular tissue) of an OCT image was considered as the domain that lumen expands 1 mm outward. Lumen and guidewire line segmentation can greatly improve the performance of characterization by implying an additional known condition during the analysis. This process is necessary because even experienced interventional cardiologists cannot always clarify the difference between the outer border of lipid tissue and the background. If the region of interest is not strictly defined, the outer border of the segmentation result will be uneven.

**Figure 4:**
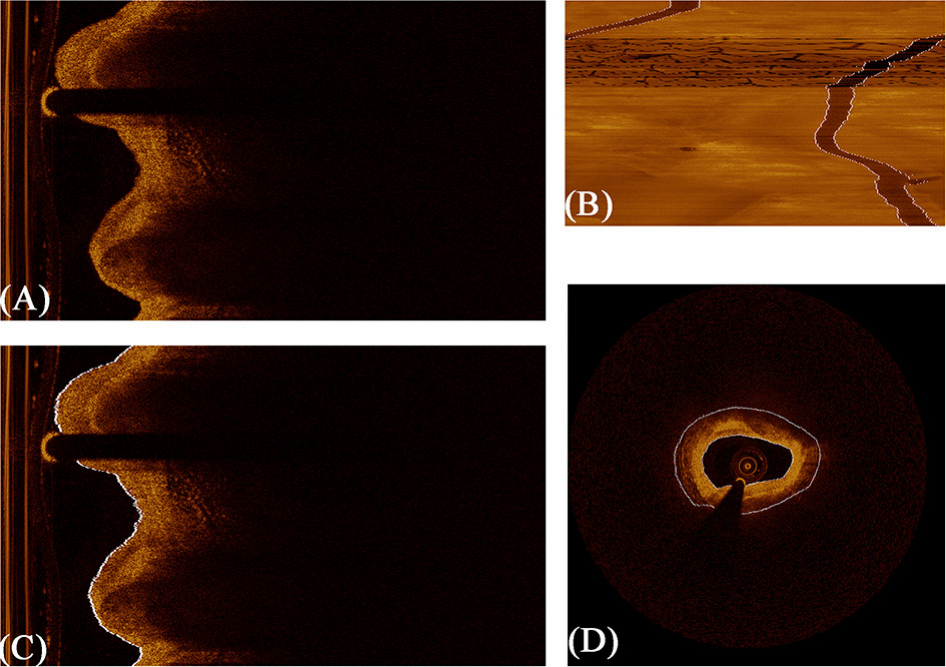
The procedure of fully automated lumen segmentation based on dynamic programming. **(A)** shows the original image in polar coordinate. **(B)** shows the concentration of average intensity of each frame; the guidewire line region became a continuous dark band traversing the whole image. **(C)** shows the lumen segmentation result with guidewire line exclusion, and **(D)** shows the regions of interest (vascular tissue, the region between the two white lines) segmented by the algorithm.

The lumen and guidewire line segmentation was based on dynamic programming, which has been proven effective and robust in Wang et al.'s research (40). The lumen boundary was detected according to the gradient in original polar image and the contour with the highest pixel value difference across the vertical axis. Guidewire always shows a long dark shallow in OCT images. Therefore, in terms of guidewire line region, the average intensity of each A-line is significantly lower. When concentrating the average intensity of all images in the whole pullback together, due to the continuity of pullbacks, the guidewire appears as a dark strip throughout the vertical axis, as shown in Figure 4B. In this condition, using dynamic programming can directly extract the guidewire of all frames in the whole pullback. On the basis of resolution of intravascular C7-XR OCT (15 μm), a constant depth of 100 pixels was utilized in our study. Figure 4 shows the whole segmentation process.

### Implementation Details

Our study is based on the deep learning toolbox in Matlab platform. Deep learning toolbox integrates multiple application and visualization tools to create and analyze deep learning architecture. It also supports the use of NVIDIA GPU, which can greatly accelerate the training process of CNN. Moreover, Matlab has excellent computing capability in matrix and is very effective in disposing data like images or feature maps.

The hyper-parameters of CNN architecture are shown in Figures 2, 3. The pooling layers uniformly applied max pooling with a stride of 1. As for optimizer selection, we chose RMSprop (41), which has been proven efficacious in many deep learning networks. The squared gradient decay factor was set to 0.9. These hyper-parameters and optimizer selection were determined according to the cross-validation results. The chosen hyper-parameters and optimizer achieved the best performance on the validation sets.

For testing time, the specific structure of convolutional output layer mentioned in section CNN Approach can accelerate the proceeding by simplifying the computation. This was realized by feeding a full image instead of individual patches as the input. The trained models can automatically convert the image under test to all label probabilities of the entire image. This testing mode can be 40 times faster than extracting patches at each pixel and predicting them individually because convolution can better deal with large-sized input compared with multiple small-sized input data. Moreover, the extracting operation is spared. Finally, as post-processing, we removed small isolated regions in the predicted results, which may be classification noise. In total, the TwoPathCNN model can provide a segmentation for each image in 2 s with the NVIDIA 1080Ti card. When using cascaded structure for prediction, it takes <3 s on average.

Because our study was focused on the characterization of plaque composition, only the prediction on the region of vascular tissue was held. After going through the trained model, the region outside the border produced in section Implementation Details was set as background by default.

## Experiments AND Results

### Evaluation Standard

F1-score (42) is an evaluation standard of classification problems, which is the harmonic average of precision and recall. Many competitions about deep learning use it as the final evaluation method. F1-score divides the classification results of models into four types as follows:

- True Positive (*TP*): Correctly predicting the target category.
- False Positive (*FP*): Incorrectly predicting other categories into the target category.
- True Negative (*TN*): Correctly predicting other categories.
- False Negative (*FN*): Incorrectly predicting the target category into other categories.

By counting the number of patches or pixels of these four types, the precision, recall, and accuracy of each category were calculated, which can be summarized as follows:

$$\begin{array}{l} precision=\frac{TP}{TP+FP}\\ \;recall=\frac{TP}{TP+FN}\\ accuracy=\frac{TP+TN}{TP+FP+TN+FN} \end{array}$$

The F1-score of each category was obtained from its precision and recall, and the general evaluation score was the average value of all the F1-scores. The details are shown in the following formula:

$$F1_{k}=\frac{2precision_{k}recall_{k}}{precision_{k}+recall_{k}}$$

$$F1-score=\frac{1}{n}\sum_{k}F1_{k}$$

Compared to the conventional accuracy evaluation standard, F1-score has a more comprehensive estimation of the CNN model. In addition, F1-score provides different indicators, which can be selectively adopted according to actual classification tasks.

### TwoPathCNN Architecture

**Figure 5:**
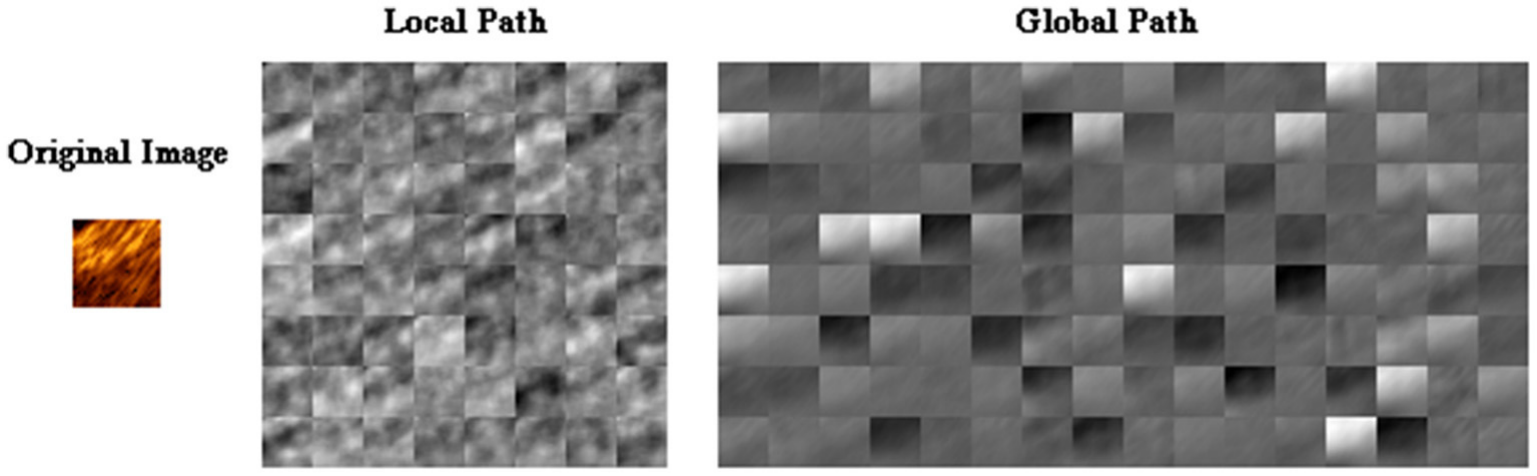
The activations of convolutional layers in the local path and the global path from a randomly selected patch. The activations can take any value, so the value is normalized to the range from 0 to 1 for visualization. Here, white pixels represent a strong positive activated region and black pixels represent a strong negative one. The channel is mostly gray if it does not activate as strongly on the original image.

To clearly illustrate the different features extracted from different paths of TwoPathCNN architecture, the activations of convolutional layers in each path is shown in Figure 5 to observe which areas activate on the patches in the convolutional layers. Although there are explicit explanations on the advanced features learned by CNN, different emphasis is indicated in these two paths. The global path mainly detects the edge information of plaques, whereas local path pays more attention on the localized texture features.

To study the effect of TwoPathCNN architecture compared to conventional linear CNN networks, we regarded the CNN model consisting of only the local path or the global path as the control group. The results of these variations are listed in Table 1, which contains the performance of TwoPathCNN, LocalPath, and GlobalPath in terms of precision and recall value of each class, the average F1-score, and the overall accuracy. According to the result of Wilcoxon signed-rank test, both F1-score and accuracy have a significant improvement compared to the control group. Moreover, the confidence interval of precision and recall on TwoPathCNN is evidently narrower than the other two CNN architectures. This indicates that TwoPathCNN is more stable and robust than the original CNN architecture because different OCT images can obtain similar performance on TwoPathCNN model. TwoPathCNN joins the local and global paths to co-adapt the features learnt from the training data, thus actualizing the promotion of capability and robustness of the CNN model.

**Table 1:** The confidence interval of performance on different CNN architectures, including precision, recall, F1-score, and accuracy.

**Architecture**	**Class**	**Precision**	**Recall**	**F1-score**	**Accuracy**
TwoPathCNN	Fibrous	0.96 ± 0.03	0.95 ± 0.03	0.86 ± 0.06^*^	0.88 ± 0.05^*^
	Calcified	0.68 ± 0.12	0.87 ± 0.06		
	Lipid	0.89 ± 0.13	0.81 ± 0.11		
LocalPath	Fibrous	0.94 ± 0.05	0.93 ± 0.05	0.81 ± 0.07	0.85 ± 0.06
	Calcified	0.63 ± 0.23	0.75 ± 0.15		
	Lipid	0.85 ± 0.12	0.77 ± 0.13		
GlobalPath	Fibrous	0.96 ± 0.04	0.87 ± 0.10	0.71 ± 0.09	0.77 ± 0.09
	Calcified	0.43 ± 0.18	0.67 ± 0.13		
	Lipid	0.80 ± 0.23	0.74 ± 0.16		

With TwoPathCNN, both F1-score and accuracy were significantly improved compared to GlobalPath and LocalPath (^*^p < 0.05). For statistical analysis, the Wilcoxon signed-rank test was performed.

### Cascaded Structure

To investigate the performance of cascaded structure, we applied the cascaded structure to TwoPathCNN architecture and compared it with the same architecture without using it. In order to maintain the consistency of the whole structure, when training the newly built dataset by concatenation, we selected the same architecture as the network that generated output comprised in the concatenated dataset (Output 1 in Figure 4). At the same time, we also investigated the impact of two-phase training method in our dataset. Two-phase training method retrains the CNN network with the unbalanced training set and keeps all the layers fixed except the output layer in the second phase. Therefore, we divided the testing performance into four groups: cascaded structure with two-phase training, cascaded structure with original training, non-cascaded structure with two-phase training, and non-cascaded structure with original training. Table 2 provides the corresponding performance results.

**Table 2:** The confidence interval of performance with or without training strategies (cascaded structure and two-phase training).

**Structure/training**	**Class**	**Precision**	**Recall**	**F1-score**	**Accuracy**
Cascaded and two-phase training	Fibrous	0.96 ± 0.03	0.95 ± 0.03	0.86 ± 0.06^*^	0.88 ± 0.05^*^
	Calcified	0.68 ± 0.12	0.87 ± 0.06		
	Lipid	0.89 ± 0.13	0.81 ± 0.11		
Cascaded and one-phase training	Fibrous	0.97 ± 0.02	0.90 ± 0.06	0.82 ± 0.07	0.86 ± 0.08
	Calcified	0.58 ± 0.15	0.94 ± 0.03		
	Lipid	0.86 ± 0.14	0.78 ± 0.15		
Non-cascaded and two-phase training	Fibrous	0.95 ± 0.04	0.89 ± 0.04	0.75 ± 0.06	0.80 ± 0.06
	Calcified	0.51 ± 0.19	0.64 ± 0.08		
	Lipid	0.77 ± 0.17	0.81 ± 0.09		
Non-cascaded and one-phase training	Fibrous	0.93 ± 0.09	0.87 ± 0.08	0.74 ± 0.07	0.80 ± 0.07
	Calcified	0.50 ± 0.18	0.69 ± 0.14		
	Lipid	0.76 ± 0.18	0.79 ± 0.11		

With cascaded structure and two-phase training, both F1-score and accuracy had the best performance (^*^p < 0.05). Cascaded structure significantly improved the performance of the segmentation (p < 0.05). For statistical analysis, the Wilcoxon signed-rank-test was performed.

As shown in Table 2, we found that cascaded structure with two-phase training achieved best performance, which was shown by the Wilcoxon signed-rank test (*p* < 0.05). Regardless of whether two-phase training was utilized or not, cascaded structure significantly improved the performance over the normal structure, with a statistically significant difference for all metrics (*p* < 0.05). However, in terms of two-phase training, two-phase training with cascaded structure had shown significant improvements on F1-score and accuracy compared to one-phase training with cascaded structure (*p* < 0.05). Without cascaded structure, the overall metrics of two-phase training were slightly increased but statistically insignificant (0.05 < *p* < 0.25). Therefore, we consider that two-phase training may bring positive effects on the segmentation results, but is not as effective as cascaded structure.

### Segmentation Results

**Figure 6:**
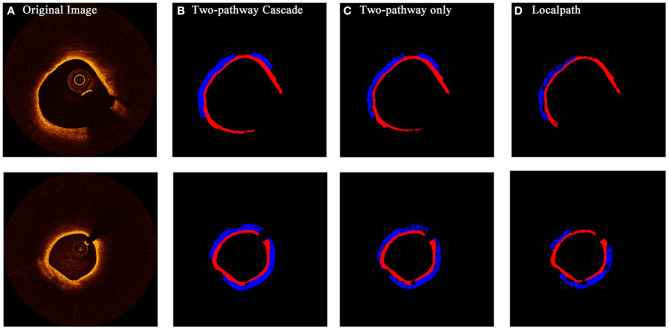
The prediction results of OCT images with fibrous caps by different types of networks. Rows **(A**–**D)** show the initial OCT image, prediction by TwoPathCNN network with cascaded structure, prediction by TwoPathCNN only, and prediction by LocalPath, respectively. Different colors represent different plaque composition: blue: lipid, red: fibrous.

**Figure 7:**
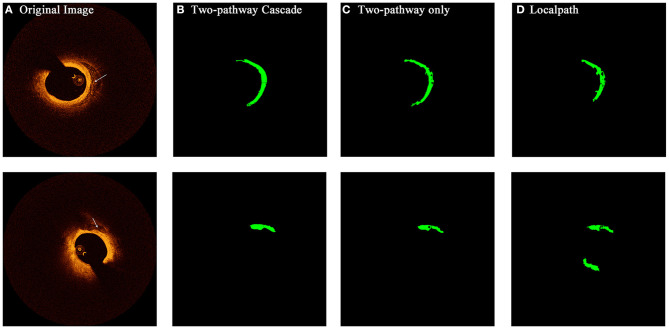
The prediction results of OCT images with calcified composition by different types of networks. Rows **(A–D)** show the initial OCT image, prediction by TwoPathCNN network with cascaded structure, prediction by TwoPathCNN only, and prediction by LocalPath, respectively. The characterized calcific composition is marked in green.

From the confidence interval of precision and recall value in each class shown in Tables 1, 2, we found that in all cases, CNN models had shown best performance in fibrous tissues, whereas the calcified tissue had a lower precision and recall coefficients compared to other tissues. From small patches, many texture features were learnt by CNN models, but location features and edge features were ignored at the same time. Fibrous tissues usually express high-intensity region in OCT images, which is significantly different from other tissues. Experts usually identify calcified tissues according to clear boundaries, but in terms of texture, the calcified tissues represent similar features with other tissue types in many cases. Although many strategies like cascaded structure were taken to add more location information into CNN models, some features were still lost in small patches. Figures 6, 7 show the prediction of different types of plaques with different networks. Compared with the results of TwoPathCNN, the cascaded structure makes the boundary of characterized plaque smoother, which is more reasonable and accurate. Whereas, the LocalPath network mis-characterized the plaque in some cases.

Table 3 shows a comparison between our CNN architectures and other published state-of-the-art methods in plaque characterization. We chose a recently published method using deep learning (29) and a machine learning method established by our group (22) for comparison. Compared with the deep learning method, although our method had a similar performance in calcified and lipid tissues, our method is able to achieve a more complicated task by segmenting the fibrous tissues. In terms of comparison with the machine learning method, our method achieved a better performance in all cases. Lambros et al. (33) used normal patch-based CNN to categorize four plaques and achieved good results in overall accuracy. However, the sensitivity is relatively low especially for calcified plaques. According to the consensus, calcified plaque contains IVOCT evidence of fibrous tissue, along with calcium that appears as a signal-poor or heterogeneous region with a sharply delineated border (43). Therefore, it is hard to characterize calcified plaques by only using texture features; thus, the normal patch-based CNN models have a poor performance. In this study, by applying cascaded structure, more information of location and border is learnt by CNN models, which can greatly improve the characterizing performance on calcified plaques. In addition, by using convolutional output layer, the computation time can be reduced.

**Table 3:** Comparison of our method with other state-of-the-art segmentation methods published previously in terms of sensitivity and specificity (—— means the author did not segment the corresponding plaques).

**Methods**		**Fibrous**	**Calcified**	**Lipid**
Our method	Sensitivity	0.95 ± 0.03	0.87 ± 0.06	0.81 ± 0.11
	Specificity	0.97 ± 0.02	0.92 ± 0.06	0.96 ± 0.03
Deep learning method	Sensitivity	——	0.85 ± 0.07	0.87 ± 0.07
	Specificity	——	0.94 ± 0.02	0.90 ± 0.05
Machine learning method	Sensitivity	0.81	0.65	0.80
	Specificity	0.91	0.91	0.88
Other patch-based method	Sensitivity	0.89	0.34	0.88
	Specificity	0.98	0.97	0.98

## Discussion

Currently, plaque characterization from OCT imaging requires trained observers, which is impractical when analyzing hundreds of pullbacks in the clinical setting. Therefore, developing an automatic algorithm for plaque characterization can effectively assist clinician in analyzing OCT images and facilitate the clinical diagnosis. We proposed a CNN-based algorithm for the automated characterization of coronary plaque compositions from OCT imaging and reformed the conventional CNN architecture according to the features of plaque composition. The results show that our CNN-based method can effectively segment different plaque compositions using trained CNN models. For each OCT image, all pixels in the region of vascular tissue are classified to one of the tissue types of atherosclerotic plaque components.

Compared to the accuracy of conventional methods using machine learning (around 81%), most architectures we mentioned in this paper can achieve this accuracy under the premise of considering a more complicated situation that an additional category besides fibrous, calcified, and lipid is taken into consideration. Methods based on texture and attenuation coefficients analyze each individual pixel with relevant intensity and texture features. This often leads to scattered noise in final prediction when the texture of tissue is uneven. Thanks to the cascaded structure, this problem has been greatly improved by providing more information on local tissue.

The algorithm was programmed in Matlab R2019b and tested in the computer with the following configuration: Windows 64-bits, CPU Intel i7 with 6 cores at 3.4 GHz, 32 GB RAM, and two NVIDIA 1080Ti cards. In machine learning methods, computational time of analysis is around 80 s per image. However, it takes only 3 s on average when using cascaded structure for prediction, which includes the lumen segmentation operation and plaque characterization. CNN methods and GPU equipment greatly accelerate the characterization. This gap in efficiency is huge especially when dealing with large datasets consisting of hundreds of OCT images.

When processing the dataset consisting of OCT imaging data, we had to exclude some images, which were hard to identify their plaque composition to prevent controversial ground truth from misleading the CNN models. This may result in error when testing on similar OCT images. Therefore, referring to segmentation results of neighboring images in the pullback and using interpolation to adjust the prediction is sometimes necessary.

OCT images *in vivo* do not have their corresponding histology slices, so the ground truth datasets were produced by trained observers *via* manual analysis. Although a series of actions were taken to reduce bias, inevitably, manual analysis of OCT images is prone to emerge error resulting in a weaker ground truth. This may affect the testing results and mislead the CNN network during the training process.

To deal with these limitations, a platform providing large amounts of OCT images and agreed validation standard can greatly improve the performance of characterization. The training sets of plaque composition require trained observers to segment substantial images manually, which is time- and labor-consuming. In addition, more observers may be required to reduce possible errors due to inter-operator variability. Lack of dataset has become a huge barrier in the promotion of automatic characterization of plaque composition. For example, accumulating evidence has shown that plaque erosion and calcified nodule are the underlying pathological causes for acute coronary events. However, due to the limitation of dataset, only a small amount of OCT images indicates the features of plaque erosion and calcified nodules, which cannot meet the requirement of CNN training. Increasing classification categories also affect the performance of CNN models especially when the training dataset is small. Given that our computational resource is limited, we selected a simple structure of CNN with only 20 layers and the input size of 33 × 33 × 8. Under the precondition of enough memory, more complex network structures can be developed to improve the performance of characterization.

In addition, although conventional OCT can provide morphological detail because of the high resolution, it also has some limitations in diagnosis. Lipid plaque by OCT is a signal-poor region with poorly delineated borders. However, the signal-poor region may represent other tissues like macrophage or intraluminal debris. Therefore, conventional OCT cannot provide a complete description of coronary artery, which may be one of the reasons why current methods to characterize plaque components has been reported with limited success. The next-generation OCT (44–46) can detect signal like unique human coronary autofluorescence signature during the imaging procedure, which may provide complementary information to that obtained by conventional OCT. In further research, auto-characterizing algorithms can be combined with these features provided by the next-generation OCT, which can improve the capacity of plaque characterization and risk assessment.

## Conclusion

We developed a novel automated algorithm based on CNN to characterize coronary atherosclerotic plaque composition from *in vivo* OCT imaging. F1-score and accuracy index were used to evaluate its performance. The experimental results show that CNN with cascaded structure can greatly improve the performance of characterization compared to the conventional machine learning methods. This method has a higher efficiency, which may be proven to be a promising practical method for future clinical practice. With further development and validation, it may be developed to be an automatic OCT-based diagnostic tool for coronary plaque characterization.

## Data Availability Statement

The original contributions presented in the study are included in the article/supplementary material, further inquiries can be directed to the corresponding author.

## Author Contributions

YY and CH contributed to the design of the study and performed the statistical analysis. BX provided the clinical OCT data and instructed the manual segmentation. YY wrote the first draft of the manuscript. ZL was in charge of the whole research and polished the manuscript. All authors contributed to manuscript revision, read, and approved the submitted version.

## Conflict of Interest

The authors declare that the research was conducted in the absence of any commercial or financial relationships that could be construed as a potential conflict of interest.
